# Identification of novel mutations in congenital afibrinogenemia patients and molecular modeling of missense mutations in Pakistani population

**DOI:** 10.1186/s12959-017-0143-3

**Published:** 2017-09-12

**Authors:** Arshi Naz, Arijit Biswas, Tehmina Nafees Khan, Anne Goodeve, Nisar Ahmed, Nazish Saqlain, Shariq Ahmed, Ikram Din Ujjan, Tahir S Shamsi, Johannes Oldenburg

**Affiliations:** 1 National Institute of Blood Diseases and Bone Marrow Transplantation, Karachi University of Bonn, ST 2/A, Block-17, Gulshan-e-Iqbal KDA scheme, 24, Karachi, Pakistan; 2 Institute of Experimental Hematology and Transfusion Medicine, Bonn, Germany; 3 University of Shieffield, Shiefield, United Kingdom; 4 Children’s Hospital, Resident, Paediatric hematology, Main Ferozpur Road, Lahore, Pakistan; 5 Liaquat university of medical and health sciences, Jamshoro, Pakistan; 6 Institute of Experimental Hematology and Transfusion Medicine, AG, FXIII Room No. 2.308 Sigmund Freud Street-25, 53127 Bonn, Germany; 7 National Institute of blood diseases and bone marrow transplantation, ST 2/A, Block-17, Gulshan-e-Iqbal KDA scheme, 24, Karachi, Pakistan; 8 Clinical Scientist and Professor of Molecular Medicine, Sheffield Diagnostic Genetics Service, Sheffield Children’s NHS Foundation Trust, Western Bank, Sheffield, S10 2TH UK; 9 Institute of Experimental Hematology and Transfusion Medicine, Sigmund Freud Street-25, 53127 Bonn, Germany

**Keywords:** Afibrinogenemia, Molecular modeling, *FGA* gene, Consanguinity, Inherited bleeding disorder

## Abstract

**Background:**

Congenital afibrinogenemia (OMIM #202400) is a rare coagulation disorder that was first described in 1920. It is transmitted as an autosomal recessive trait that is characterized by absent levels of fibrinogen (factor I) in plasma. Consanguinity in Pakistan and its neighboring countries has resulted in a higher number of cases of congenital fibrinogen deficiency in their respective populations. This study focused on the detection of mutations in fibrinogen genes using DNA sequencing and molecular modeling of missense mutations in all three genes [Fibrinogen gene alpha (FGA), beta (FGB) and gamma (FGG)] in Pakistani patients.

**Methods:**

This descriptive and cross sectional study was conducted in Karachi and Lahore and fully complied with the Declaration of Helsinki. Patients with fibrinogen deficiency were screened for mutations in the Fibrinogen alpha (*FGA*), beta (*FGB*) and gamma (*FGG*) genes by direct sequencing. Molecular modeling was performed to predict the putative structure functional impact of the missense mutations identified in this study.

**Results:**

Ten patients had mutations in *FGA* followed by three mutations in *FGB* and three mutations in *FGG*, respectively. Twelve of these mutations were novel. The missense mutations were predicted to result in a loss of stability because they break ordered regions and cause clashes in the hydrophobic core of the protein.

**Conclusions:**

Congenital afibrinogenemia is a rapidly growing problem in regions where consanguinity is frequently practiced. This study illustrates that mutations in *FGA* are relatively more common in Pakistani patients and molecular modeling of the missense mutations has shown damaging protein structures which has profounding effect on phenotypic bleeding manifestations in these patients.

## Background

Hemostasis is the normal physiological response that prevents blood loss following vascular injury. It is dependent on an intricate series of events involving platelets and specific coagulation factors. Inherited bleeding disorders can be grouped into abnormalities of primary and secondary hemostasis. Fibrinogen (Factor I) deficiency can originate from congenital or acquired causes. Congenital afibrinogenemia (*OMIM #202400*) is a rare coagulation disorder that was first described in 1920 [1]. It is as a recessive autosomal inherited trait characterized by the absence of fibrinogen (factor I) in plasma [2]. The disease has a worldwide prevalence of 1–2 per million in the general population [3]. Fibrinogen is a 340 KDa hexameric protein of hepatic origin with multiple functions including roles in platelet aggregation and platelet plug formation and is an acute phase reactant [4]. It is secreted as zymogen similar to all other clotting factors and needs to be activated prior to its participation in the coagulation cascade. It consist of three pairs (Aα, Bβ and Gγ) of polypeptide chains [5] encoded by three genes (*FGA, FGB and FGG*) clustered in a region of approximately 50 kb on chromosome 4q28-q31 [6, 7]. The normal plasma levels of fibrinogen are 4 g/l [8, 9] and its half-life is approximately 100 h/4 days [10]. The main role of fibrinogen in hemostasis is to strengthen the platelet plug by converting into its polymeric insoluble form called fibrin by thrombin [11]. The fibrin meshwork traps red blood cells and platelets to form a plug which stops bleeding from site of injury. The absence of fibrinogen may result in excessive blood loss after a trauma. Moreover spontaneous bleeding events can occur. Fibrinogen defects are classified as quantitative (Hypofibrinogenemia and Afibrinogenemia, depending upon the partial or complete absence of fibrinogen) or qualitative (Dysfibrinogenemia and Hypodysfibrinogenemia) [12]. The most common symptom associated with fibrinogen deficiency is umbilical stump bleeding with other secondary bleeding manifestations including epistaxis, gum bleeding, cutaneous bleeding, muscle hematoma and haemarthrosis [13].

Congenital fibrinogen deficiency is considered as rare coagulation disorder but its incidence is growing higher in those regions where consanguineous partnerships are common [14, 15]. Pakistan is the country with high ratio of consanguinity resulting in increasing numbers of rare inherited bleeding disorders including congenital afibrinogenemia. Our focus was to identify the mutations, assess the possible structure functional impact of affected protein by using molecular modeling/silico analysis tools. In addition to this, the study also encompasses the insight for possible mutational spectrum in frequently involved fibrinogen gene which may contribute for future prenatal diagnosis of carriers of these defects in Pakistani population.

## Methods

### Patient inclusion and exclusion criteria

This study, involving human subjects, was performed according to the Declaration of Helsinki, 1975, revised in 2000, and was approved by the relevant institutional Ethical Committee. Patients with congenital afibrinogenemia i.e. absent or undetectable levels of fibrinogen antigen (0–0.1 g/dl) and it activity in plasma were selected for this study. These low levels excluded acquired causes of fibrinogen deficiency, such as liver disease and consumptive coagulopathies, leukemia or other factor deficiencies. Patients from across Pakistan were recruited from centers including Karachi (Sindh) and Lahore (Punjab). A written informed consent was taken from patients and guardians incase of minor. Sampling was performed independent of sex or age. A comprehensive questionnaire was completed containing information about the patient’s demographics and disease symptoms. A diagnosis was made on the basis of history and quantitative analysis. All subjects were registered at Hemophilia Society of Pakistan. Samples from all centers were collected and initially processed and saved at the National Institute of Blood Diseases (NIBD) for coagulation profile, biochemistry tests including liver profile and viral markers. DNA sequencing was performed in NIBD genome department, Karachi.

### Sample collection and lab assays

Blood samples from patients were collected in 3.2% sodium citrate for coagulation profile in serum (RST) for biochemistry analysis, including liver profile and viral profile, (HBsAg, Anti HCV and HIV) and in K_2_EDTA for complete blood count and DNA extraction for amplification and sequencing. All sampling was performed with supportive infusion of cryoprecipitate to avoid bleeding. Platelet-poor plasma was collected by centrifugation of citrate tubes at 4000×g for 10 min and coagulation profile was performed, including PT, APTT and fibrinogen assay, using the Clauss method. Liver function tests (direct and indirect bilirubin, ALT, AST and alkaline phosphatase) and viral markers (HBsAg, anti HCV and HIV) were performed to exclude any acquired cause of afibrinogenemia.

Genetic analysis was performed after isolation of genomic DNA using standard protocols, exons and intron-exon junctions of the fibrinogen genes were amplified by polymerase chain reaction [16] and sequenced [17] as previously described.

### Pathogenecity scoring

Pathogenecity scoring was done by five prediction tools to predict the possible structure functional impact of affected protein in identified novel missense mutations. The prediction software tool Poly-phen2 (polymorphism phenotyping v2), (http://genetics.bwh.havard.edu/pph2/ accessed on 20th April 2015) was used to assess the possible impact of substitution on structure and function in human SNPs (Single nucleotide polymorphism). MUPRO (predictions of protein stability changes upon mutations), (http://mupro.proteomics.ics.uci.edu/ accessed on 20th April 2015) utilizes an SVM (support vector machines) model to predict the changes in stability as a result of single-site mutations, primarily from sequential information, and optionally provided structural information. The result only predicts whether the alteration in single amino acid will lead to destabilization or not. MUPRO predictions are reported with the confidence score (C score). A positive score indicates higher stability whereas a negative score shows the mutation decreases the protein stability (http://mupro.proteomics.ics.uci.edu/ accessed on 20th April 2015). SNP&GO (Single nucleotide polymorphism and GO terms, http://snps.biofold.org/snps-and-go accessed on 20th April 2015). SIFT (Sorting Intolerant from Tolerant, http://sift.jcvi.org accessed 20th April 2015) are algorithms which predict whether an amino acid substitution will affect protein function based on sequence homology and the physical properties of amino acids. A SIFT score of less than 0.05 is predicted to be deleterious. A substitution with a score greater than or equal to 0.05 is predicted to be tolerated (http://www.exeterlaboratory.com/molecular-genetics/). Provean (http://provean.jcvi.org/about.php) accessed on 27th January 2015) has the default threshold of −2.5 that means if the score of a variant is equal or below this threshold then the mutation is said to be deleterious and if the threshold is above −2.5, the score of variant is said to have neutral effects. Protein accession numbers were provided by Uniprot (Universal Protein Resource, http://www.uniprot.org/) and wild type color fasta sequence was first accessed (http://pga.gs.washington.edu/data/fga/fga.Colorfasta.html) on 27th January 2015 and later on 20th April 2015.

### Structural analysis of novel missense mutations using molecular modeling

Among the six reported novel missense mutations from this study, four mutations were located in an area of the alpha chain that has no resolved crystal/NMR-based structure (Nuclear magnetic Resonance). Thus, to assess the putative structural effect of these mutations, we modeled this region on the ITASSER (Iterative Threading ASSEmbly Refinement) threading modeling server (http://zhanglab.ccmb.med.umich.edu/I-TASSER/; accessed on 12th November 2014). The model for this region was then joined to the remaining beta chain for which the structure has already been determined and submitted in the protein structure database (PDB file ID: 3GHG; 2.9 Å resolution). Model joining was performed by replacing the last two amino acid residues common to the model and the crystal structure (PDB file ID: 3GHG; chain A) (PDB: Protein Data Base, ID: Identity, 3ghg is a 4-character unique identifier of every entry in the Protein Data Bank) downloaded from the protein structure database (http://rcsb.org/pdb/home/home.do;) accessed 20th November 2014) to maintain the dihedral angles for the full model at the point of joining the same. The complete model was refined by a short solvated simulation lasting 500 ps as described in Krieger et al., 2004 (Force field: Yamber3, periodic boundary conditions, temperature: 298 K, water density: 0.997 g/L, pH: 7.4). The local neighborhood of the wild type residue corresponding to the reported mutation was investigated to establish a logical hypothesis for the effect of the mutation. An additional one missense mutations (p.Trp432Arg) in the beta chain lies on the structurally resolved region of the PDB file 3GHG; chain B). Similarly the local molecular environment for this wild-type residue was also inspected. All structural analysis and image rendering were performed with YASARA (Yet Another Scientific Artificial Reality Application) version 12.8.6 (www.yasara.org/).

## Results

Mutations were identified in all 13 patients. The major bulk of identified mutations is present in *FGA* gene which tends to be the most frequently occurring mutation site in our study population. Ten patients who have mutations in *FGA* gene are individual unrelated probands.

Mutations in *FGB* gene are less frequent as compared to *FGA*.

In *FGA* gene, eight mutations were identified as novel and the remaining two were reported mutations. Eight novel mutations include five missense, one nonsense and two frameshift mutations including homozygous and a compound heterozygous frameshift mutation. The two nonsense mutations in *FGA* are reported in literature. There is one more mutation with reported status in proband (C3). This patient had compound heterozygous mutation with frameshift as novel mutation and nonsense as reported.

We identified three mutations in *FGB* including one novel missense mutation (C9) and two homozygous nonsense mutations reported in siblings.

The *FGG* gene mutations are the rarest of all three fibrinogen genes. We detected three novel mutations including two similar nonsense mutations in siblings and one frameshift mutation in unrelated proband in different exons of *FGG* gene (Table 1).

**Table 1:** Genotypic expression of mutations in fibrinogen gene (*FGA, FGB & FGG*)

IP #	Gene	Exon	Mutation	Amino Acid change	Zygosity	Mutation type	Reported/Novel
C1	***FGA***	1	c.24C > A	p.Cys8^a^	Homozygous	Nonsense	Ref [23] ^€^
C2		2	c.143_144 del AA	p.Lys(AAA)48Arg fs9^a^	Compound Heterozygous	Frame shift	Novel mutation
C3		5	c.846delG	p.Gln282Thr fsx83^a^	Compound Heterozygous	Frame shift	Novel mutation
		4	c.385C > T	p.Arg129^a^	Homozygous	Nonsense	Ref [24] ^€^
C4		4	c.385 C > T	p.Arg129^a^	Homozygous	Nonsense	Ref [24] ^€^
C5		5	c.598C > T	p.Gln183^a^	Homozygous	Nonsense	Novel mutation
C6		5	c.904C > G	p.Pro302Ala	Homozygous	Missense	Novel mutation
C7		5	c.913A > G	p.Thr 305 Ala	Homozygous	Missense	Novel mutation
C8		5	c.992A > G	p.Thr331Ala	Homozygous	Missense	Novel mutation
C9		5	c.992A > G	p.Thr331Ala	Homozygous	Missense	Novel mutation
C10		5	c.974A > G	p.Ser325Gly	Homozygous	Missense	Novel mutation
C11A	***FGB***	2	c.141 > T	p.Arg47^a^	Homozygous	Nonsense	Ref [25] ^€^
C11B		2	c.141C > T	p.Arg47^a^	Homozygous	Nonsense	Ref [25] ^€^
C9		8	c.1294 T > A	p.Trp 432Arg	Homozygous	Missense	Novel mutation
C12	***FGG***	2	c.120_126dupTTCTTCA	TTCTTCA	Homozygous	Frame shift	Novel mutation
C13A		4	c.361A > T	p.Lys121^a^	Homozygous	Nonsense	Novel mutation
C13B		4	c.361A > T	Lys121^a^	Homozygous	Nonsense	Novel mutation

Identified novel and reported mutations in three genes of fibrinogen. The letter A and B with patient code designate the sibling status. € (reported mutation,) c (complimentary deoxyribonucleic acid), A (adenine), T (thymine), C (cytosine), G (guanine), Lys (lysine), Arg (arginine), Tyr (tyrosine), Pro (proline), Trp (tryptophan), Thr (threonine), Gln (glycine), Cys = cystine, fs = frame shift, ^a^ stop codon number, *FGA* (fibrinogen Aα-chain gene), *FGB* (fibrinogen Bβ-chain gene), *FGG* (fibrinogen GƔ-chain gene

All patients had markedly absent fibrinogen levels (0 g/l) and prolonged PT >120 s and APTT >180 s (Table 2).

**Table 2:** Assessment of coagulation markers and bleeding scores with consanguinity/ethnicity

IP#	Fibrinogen Level (g/l)	Thrombin Time (Sec)	Prothrombin Time (Sec)	Activated partial thromboplastin Time (aPTT)(Sec)	Bleeding Score	Consanguinity	Interfamilial Relation	Ethnic Origin
C1	0.01	23	>120	>180	20	positive	Unrelated	Urdu Speaking
C2	0.02	24	>120	>180	21	positive	Unrelated	Punjabi
C3	0	33	>120	>180	22	positive	Unrelated	Punjabi
C4	0.1	24	>120	>180	17	positive	Unrelated	Urdu Speaking
C5	0.02	31	>120	>180	20	positive	Unrelated	Sindhi
C6	0.01	25	>120	>180	20	positive	Unrelated	Urdu speaking
C7	0.02	29	>120	>180	22	positive	Unrelated	Sindhi
C8	0.0	30	>120	>180	20	positive	Unrelated	Sindhi
C9	0.0	32	>120	>180	22	positive	Unrelated	Punjabi
C10	0.01	25	>120	>180	16	positive	Unrelated	Punjabi
C11A	0.02	28	>120	>180	18	positive	**	Punjabi
C11B	0.01	24	>120	>180	16	positive		Punjabi
C12	0.0	30	>120	>180	21	positive	Unrelated	Punjabi
C13	0.01	24	>120	>180	20	positive	Unrelated	Punjabi
C14	0.0	26	>120	>180	21	positive	Unrelated	Punjabi
C15A	0.02	24	>120	>180	20	positive	**	Punjabi
C15B	0.01	25	>120	>180	21	positive		Punjabi

Shows the individual test values of PT, aPTT and fibrinogen (Claus Method), consanguinity and the relationship status. Bleeding score calculated, Tosetto et al. [26]. ** Siblings, *NA* not available, *s* (seconds). The fibrinogen levels in all patients were found to be equal to or lower than 0.1 g/l (Normal Range 2-4 g/dl), PT more than 120 s (Normal Range 9–11 s) aPTT more than 180 s (Normal Range 24–27 s) and prolonged thrombin time (normal range 10–13 s). Ethnicity explains the frequency of majorly affected, thickly populated and largest province of Pakistan (Punjab)

### Structural analysis of novel missense mutations using molecular modeling

#### A) alpha chain missense mutations

All four novel missense mutations from the α-chain reported in this study were present in a region (residues 220–860) of the α-chain, which had no resolved/known crystal structure. The region surrounding the reported mutations (residues 300–400) was relatively poorly conserved with most of it missing from some fibrinogen homologues.

**Fig. 1:**
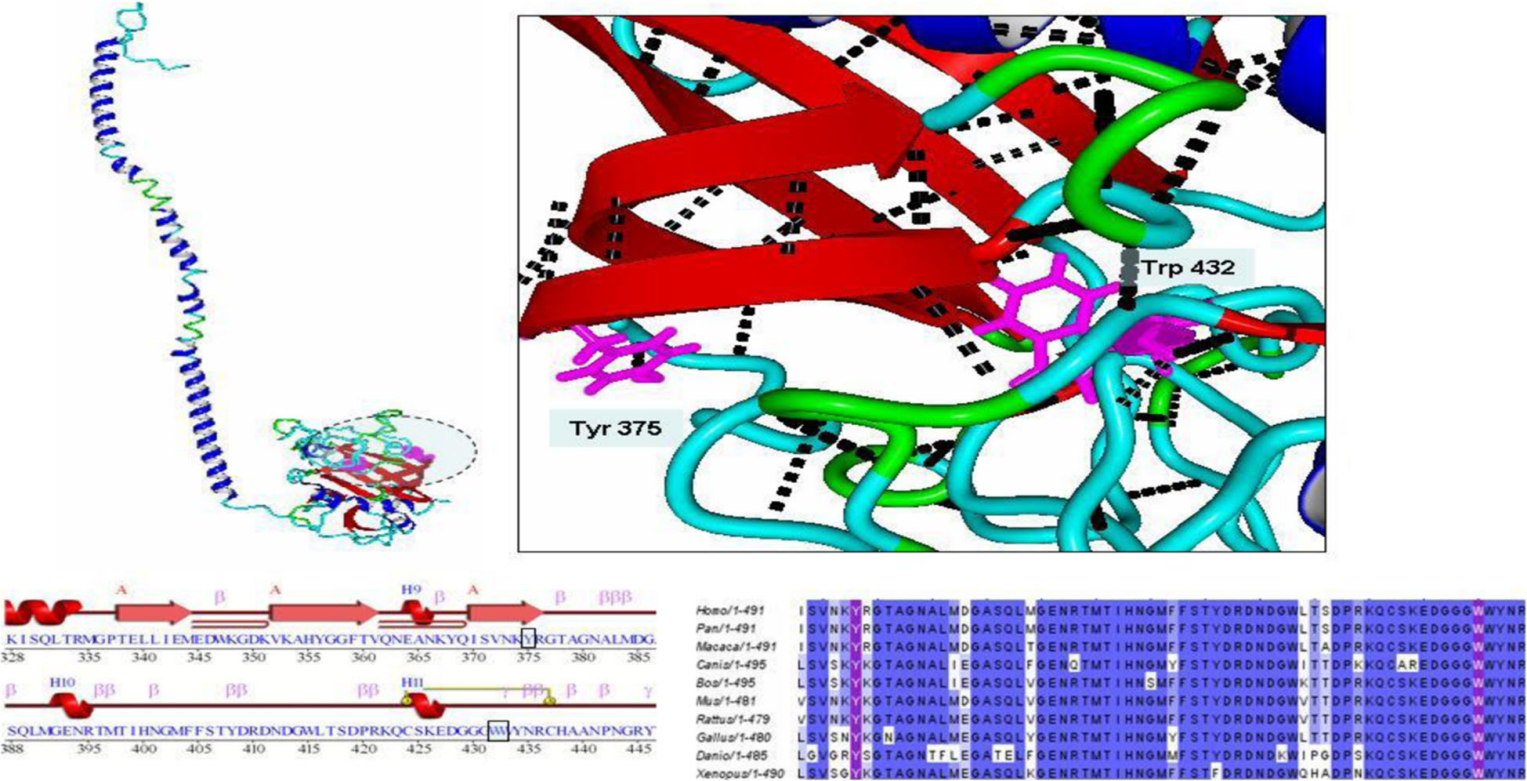
Molecular Remodeling of a missense mutation in *FGA*

Among the mutated residues, p.Pro302 was present in all homologues, which contained this part. The p.Ser325 residue was also conserved in all homologues with the exception of *Musmusculus,* where it was substituted by an Asn. The two Thr residues, p.Thr302 and p.Thr331, were relatively variable and substituted by Ser or Asp in a few homologues. Only in the homologue from *Canis lupus familiaris* was one of the Thr residues (p.Thr302) observed to be substituted by an Ala residue, which has been reported as a mutated residue for both Thr residues in our study. Modeling of this region showed that this region could be split into two central cores, each of which is organized as a beta sheet surrounded by flexible coils (Fig. 1). The two cores are connected by a central long helix. The first core, apart from being surrounded by flexible coils, also contains a few short helices. Three of the four reported mutated residues were located on these short helices with the exception that p.Ser325 is located on a short loop connecting one of the short helices to the central core. The residues p.Pro302, p.Thr305 and p.Thr331 are partially buried, with the p.Pro302 and p.Thr305 side chains oriented toward the central core beta sheets. The residues p.Pro302 and p.Thr305 participate in intra-helical hydrogen bonds with each other and with p.Ser399 and p.Arg308, respectively. The residue p.Thr331 lies at the edge of a short helix and also participates in intra-helical hydrogen bonding (p.Gly327). Interestingly, within the fold on which all four of these mutations reside, lysine residue p.Lys322 is known to be cross-linked to ∝ − 2 antiplasmin proteins and a glutamine residue, p.Gln347, which participates in inter-chain cross-links during clot formation.

#### B) Beta chain missense mutations

**Fig. 2:**
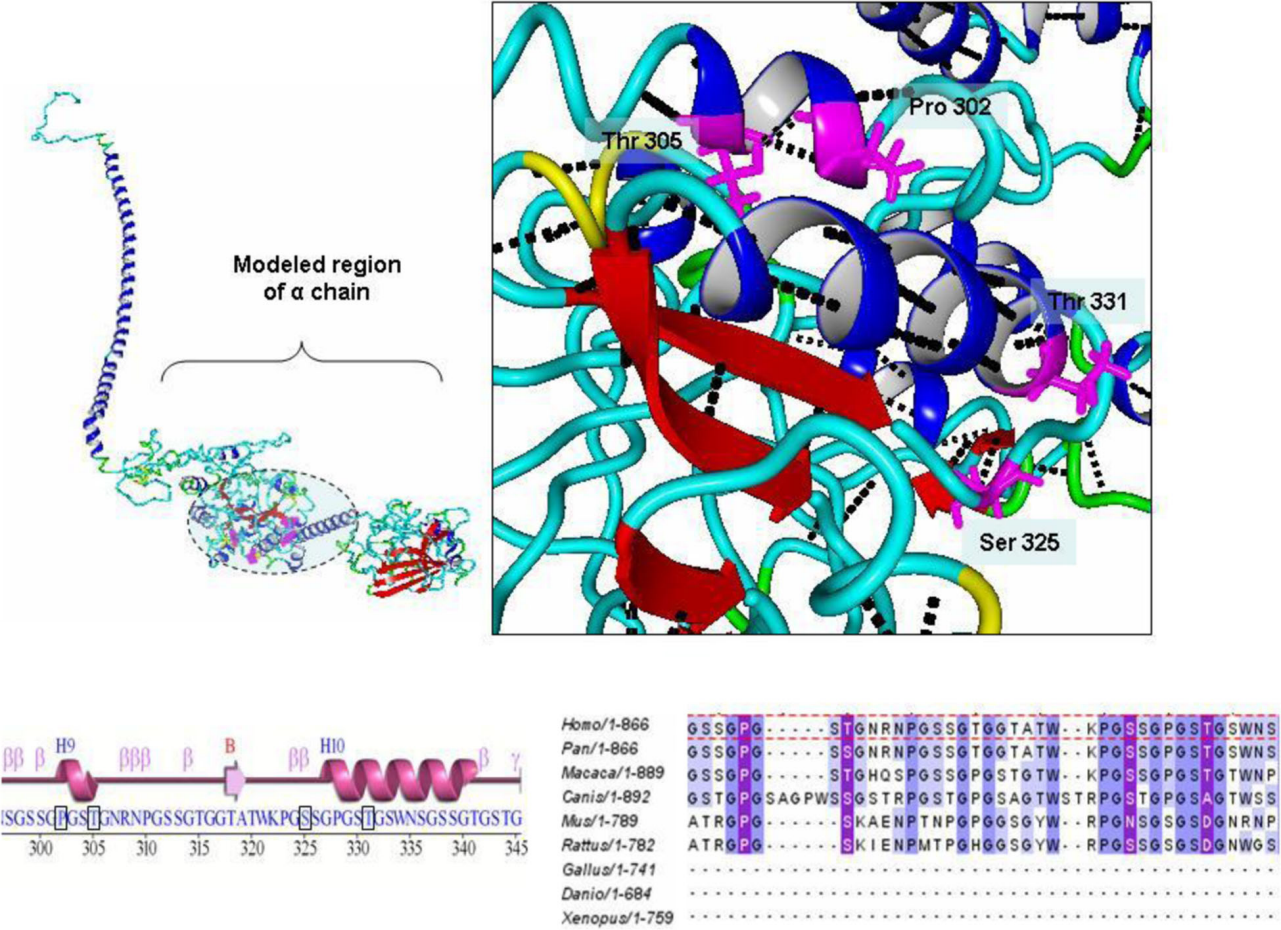
Molecular Remodeling of a missense mutation in *FGB*

The one novel missense mutations (p.Trp432Arg) reported in the chain occurs in a highly conserved region. (Fig. 2).

The residues are completely conserved in homologues that have been used for the present alignment. The p.Trp432 residue lies completely in the densely packed hydrophobic core of the C-terminal region of chain. This densely packed hydrophobic core consists of a number of other aromatic acids, which are in close proximity to p.Trp432 (p.His400 and p.His438, p.Trp433 and p.Tyr434). The p.Trp432 residue hydrogen bond contacts with p.Tyr434 and p.Ser406.

### Pathogenecity score

Pathogenecity scoring of six novel missense mutations identified in *FGA* and *FGB* was done on five different pathogenicity scoring software (Table 3). Out of five missense mutations of *FGA*, two mutations were found to have damaging effect and decreased protein stability calculated by two different softwares (MUPRO and Provean). Other software didn’t show the deleterious effect for the same two mutations identified in two unrelated proband. In *FGB* gene the missense mutation was found to be damaging or deleterious and showed decreased structure stability. The damaging effect and lack of protein stability in structure may lead to the bleeding manifestations in patients which can vary from mild to severe bleeding.

**Table 3:** Pathogenicity score of missense mutations

Missense Mutations	Polyphen- 2		Provean		MUpro		SNP&GO		Sift	
	Score	prediction	Score	prediction	SVM score	Protein structure stability	Score	Prediction	Score	Prediction
p.Pro302Ala	0.028	Benign	−4.257	Deleterious	−0.797	Decrease stability	(0.4)	Neutral	0.00	Benign
p.Thr 305 Ala	0.00	Benign	−0.387	Neutral	0.134	Increase stability	(0.05)	Neutral	0.00	Benign
p.Thr331Ala	0.025	Benign	−1.100	Neutral	0.122	Increase stability	(0.03)	Neutral	0.00	Benign
p.Thr331Ala	0.025	Benign	−1.100	Neutral	0.122	Increase stability	(0.03)	Neutral	0.00	Benign
p.Ser325Gly	0.014	Benign	−2.331	Neutral	−0.063	Decrease stability	(0.1)	Neutral	0.00	Benign
p.Trp 432Arg	1.00	Damaging	−12.18	Deleterious	−0.411	Decrease stability	(0.8)	Disease	Na	Na

Pathogenecity of missense mutations was calculated by five different softwares to check for the protein structure stability and deleterious effects. *Na* not available

## Discussion

Fibrinogen deficiency is a rare inherited bleeding disorder that is characterized by two subtypes of either reduced or completely absent levels of fibrinogen in the blood [18]. *FGA* is documented as the most affected gene in literature [19, 20]. We have found the larger chunk of mutations in *FGA* gene in our set of data. A total of 169 mutations in fibrinogen are reported on the Human Gene Mutation Database (http://www.hgmd.cf.ac.uk/ac/index.php) date accessed August 12, 2014). Consanguinity involving first and second cousin marriages is accelerating the spread of disease in areas such as Pakistan, Iran, the Middle East, China and the far Middle East, including Turkey, in societies where consanguinity is frequent. The spectrum of causative mutations for afibrinogenemia is interesting as *FGA* appears to stand out from the two other fibrinogen genes [21]. The predominant inheritance pattern was homozygous with a high proportion of nonsense mutations followed by missense mutations in our study results. A frame shift mutation (p.Glu262AspfsX158) in FGA exon 5 reported in one study is predicted as truncated polypeptide. It is associated with exceptionally long stretch of abnormal residues in homozygous patient with congenital afibrinogenemia [22]. Frameshift mutation (p.Gln282Thr fsx83*) and (p. Lys (AAA) 48Arg fs9*) are the novel compound heterozygous mutations which have manifested deletions along with frameshift defects. The bleeding phenotype is severe as these mutations worsen the symptoms due to combined effect of compound mutation and truncation of polypeptide chain.

Three missense mutations (Pro302Ala, Thr305Ala and Thr331Ala) in the alpha chain reside on short helices surrounding a central beta sheet core. All these mutations are non-conservative in nature, i.e., the Pro302Ala substitution results in the replacement of a rigid imino group with a smaller, more flexible residue, and the Thr305Ala and Thr302Ala substitutions result in the replacement of polar side chains by smaller but hydrophobic side chains. In addition, the introduction of alanine in these regions will most likely disrupt some of the intra-helical hydrogen bonds, thereby breaking the helical structure surrounding the central core. Because these short helices provide order and stability around an otherwise disordered coiled-coil region, their disruption might result in a loss of stability for this region and the alpha chain. The third mutation in this chain, Ser325Gly, is also non-conservative, i.e., it results in the substitution of a polar residue to a very small and flexible Gly residue. Because the wild-type residue already lies on the flexible loop, the introduction of a small residue will make this region more disordered and therefore unstable. Moreover, because all four missense mutations belong to a fold of the alpha chain that might be interacting with Factor XIII (this fold also contains the Lys and Gln residues that participate in interchain cross linking and cross linking to alpha 2- antiplasmin), conformational changes induced by these mutations on this fold might interfere with the interaction of fibrinogen alpha chain with Factor XIII. The one beta chain missense mutation resides on a highly conserved region of the beta chain, most likely because many of the residues of this region contribute to the stability of its densely packed hydrophobic core. The p.Trp432Arg substitution occurs in the middle of the hydrophobic core. The introduction of a large polar, positively charged residue instead of a hydrophobic aromatic one would destabilize the hydrophobic core of this region. Thus, the mutation affects the stability of the beta chain by disrupting its C-terminal hydrophobic core.

## Conclusions

Rare inherited bleeding disorder specifically congenital afibrinogenemia has a growing incidence especially in regions like Pakistan where consanguinity factor is strongly present. Our study is purely based on Pakistani patients of congenital afibrinogenemia. It has shown the frequently affected gene *FGA* in our set of patients. We have documented the pathogenicity scores for missense mutations as a description for protein molecule stability and functional defects. We have also performed molecular modeling to see the structural defects and damages and their impact on the clinical manifestation of patients. In this way the genotype well correlated with phenotype of these patients.

## Abbreviations

Ala: Alanine
ALT: Alanine transaminase
Anti-HCV: Anti hepatitis C antibodies
aPTT: Activated partial thromboplastin time
Arg: Arginine
Asn: Asparagine
AST: Aspartate transaminase
C score: Confidence score
EDTA: Ethylenediaminetetraacetic acid
FGA: Fibrinogen gene alpha
FGB: Fibrinogen gene beta
FGG: Fibrinogen gene gamma
HBsAG: Hepatitis B surface antigen
HIV: Human immunodeficiency virus
ITASSER: Iterative threading assembly refinement
KDa: Kilo daltons
Poly-phen2: Polymorphism phenotyping v2
Pro: Proline
Provean: Protein variation effect analyzer
PT: Prothrombin time
SIFT: Sorting intolerant from tolerant
SNP: Single nucleotide polymorphism
SNP&GO: Single nucleotide polymorphism and GO terms
SVM: Support vector machine
Trp: Tryptophan
TT: Thrombin time
UniProt: Universal protein resource
YASARA: Yet another scientific artificial reality application

## References

[1] Peyvandi F, Haertel S, Knaub S, Mannucci P (2006). Incidence of bleeding symptoms in 100 patients with inherited afibrinogenemia or hypofibrinogenemia. Journal of Thrombosis and Haemostasis..

[2] Acharya S, Dimichele D (2008). Rare inherited disorders of fibrinogen. Haemophilia..

[3] Neerman-Arbez M, De Moerloose P, Bridel C, Honsberger A, Sconborner A, Rossier C, Peerlinck K, Claeyssens S, Di Michele D, D'oiron R, Dreyfus M, Laubriat-Bianchin M, Dieval J, Antonarakis SE, Morris MA (2000). Mutations in the fibrinogen Aa gene account for the majority of cases of congenital afibrinogenemia. Blood..

[4] Janciauskiene S, Welte T, Mahadev R. Acute Phase Proteins: Structure and Function Relationship. Acute Phase Proteins - Regulation and Functions of Acute Phase Proteins. 2011;.

[5] Neerman-Arbez M, De Moerloose P, Honsberger A, Parlier G, Arnuti B, Biron C, Borg J, Eber S, Meili E, Peter-Salonen K, Ripoll L, Vervel C, d'Oiron R, Staeger P, Antonarakis SE, Morris MA (2001). Molecular analysis of the fibrinogen gene cluster in 16 patients with congenital afibrinogenemia: novel truncating mutations in the FGA and FGG genes. Human genetics..

[6] Anwar M, Iqbal H, Gul M, Saeed N, Ayyub M (2005). Congenital afibrinogenemia: report of three cases. Journal of Thrombosis and Haemostasis..

[7] Neerman-Arbez M, De Moerloose P (2007). Mutations in the fibrinogen gene cluster accounting for congenital afibrinogenemia: an update and report of 10 novel mutations. Human Mutation.

[8] Asselta R, Duga S, Tenchini M (2006). The molecular basis of quantitative fibrinogen disorders. Journal of Thrombosis and Haemostasis..

[9] Grieninger G, Lu X, Cao Y, Fu Y, Kudryk BJ, Galanakis DK, Hertzberg KM (1997). Fib420, the novel fibrinogen subclass: newborn levels are higher than adult. Blood..

[10] Fang Y, DAI B, WANG X, FU Q, Dai J, Xie F, CAI X, WANG H, WANG Z (2006). Identification of three FGA mutations in two Chinese families with congenital afibrinogenaemia. Haemophilia..

[11] Asselta R, Duga S, Simonic T, Malcovati M, Santagostino E, Giangr EP, Mannucci P, Tenchini M (2000). Afibrinogenemia: first identification of a splicing mutation in the fibrinogen gamma chain gene leading to a major gamma chain truncation. Blood..

[12] Casini A, Neerman-Arbez M, Ariëns R, de Moerloose P (2015). Dysfibrinogenemia: from molecular anomalies to clinical manifestations and management. Journal of Thrombosis and Haemostasis..

[13] Asselta R, Spena S, Duga S, Peyv IF, Malcovati M, Mannucci P, Tenchini M (2002). Analysis of Iranian patients allowed the identification of the first truncating mutation in the fibrinogen Bbeta-chain gene causing afibrinogenemia. Haematologica.

[14] Wu S, Wang Z, Dong N, Bai X, Ruan C (2005). A novel nonsense mutation in the FGA gene in a Chinese family with congenital afibrinogenaemia. Blood coagulation & fibrinolysis..

[15] Sumitha E, Jayandharan G, Arora N, Abraham A, David S, Devi GS, Shenbagapriya P, Nair SC, George B, Mathews V, Chandy M, Viswabandya A, Srivastava A (2013). Molecular basis of quantitative fibrinogen disorders in 27 patients from India. Haemophilia..

[16] Tyrrell DAJ (1997). Polymerase Chain Reaction. BMJ.

[17] Sanger Sequencing Method | Thermo Fisher Scientific [Internet]. Thermofisher.com. 2017 [cited 2 Febuary 2017]. Available from: https://www.thermofisher.com/pk/en/home/lifescience/sequencing/sanger-sequencing/sanger_sequencing_method.html

[18] Korte W, Poon M, Iorio A, Makris M (2017). Thrombosis in Inherited Fibrinogen Disorders. Transfusion Medicine and Hemotherapy..

[19] Neerman-Arbez M (2003). Prenatal diagnosis for congenital afibrinogenemia caused by a novel nonsense mutation in the FGB gene in a Palestinian family. Blood..

[20] VU D, NEERMAN-ARBEZ M (2007). Molecular mechanisms accounting for fibrinogen deficiency: from large deletions to intracellular retention of misfolded proteins. Journal of Thrombosis and Haemostasis..

[21] Robert-Ebadi H, De Moerloose P, El Khorassani M, El Khattab M, Neerman-Arbez M (2009). A novel frameshift mutation in FGA accounting for congenital afibrinogenemia predicted to encode an aberrant peptide terminating 158 amino acids downstream. Blood Coagulation & Fibrinolysis..

[22] Levrat E, Aboukhamis I, de Moerloose P, Farho J, Chamaa S, Reber G (2011). A novel frameshift mutation in FGA (c.1846 del A) leading to congenital afibrinogenemia in a consanguineous Syrian family. Blood Coagulation & Fibrinolysis..

[23] Asselta R, Platè M, Robusto M, Borhany M, Guella I, Soldà G (2014). Clinical and molecular characterisation of 21 patients affected by quantitative fibrinogen deficiency. Thrombosis and Haemostasis..

[24] Palermo P, Barbados B, Asselta R, Duga S, Tenchini M (2006). The molecular basis of quantitative fibrinogen disorders. Journal of Thrombosis and Haemostasis..

[25] Sheen C, Brennan S, Jabado N, George P (2006). Fibrinogen Montreal: a novel missense mutation (A alpha D496N) associated with hypofibrinogenemia. Thrombosis and haemostasis..

[26] Tosetto A, Rodeghiero F, Castaman G, Goodeve A, Federici A, Batlle J, Meyer D, Fressinaud E, Mazurier C, Goudemand J, Eikenboom J, Schneppenheim R, Budde U, Ingerslev J, Vorlova Z, Habart D, Holmberg L, Lethagen S, Pasi J, Hill F, Peake I (2006). A quantitative analysis of bleeding symptoms in type 1 von Willebrand disease: results from a multicenter European study (MCMDM-1 VWD). J Thromb Haemost..

