# Correction to: Identification of novel mutations in congenital afibrinogenemia patients and molecular modeling of missense mutations in Pakistani population

**DOI:** 10.1186/s12959-019-0193-9

**Published:** 2019-04-04

**Authors:** Arshi Naz, Arijit Biswas, Tehmina Nafees Khan, Anne Goodeve, Nisar Ahmed, Nazish Saqlain, Shariq Ahmed, Ikram Din Ujjan, Tahir S. Shamsi, Johannes Oldenburg

**Affiliations:** 1 National Institute of Blood Diseases and Bone Marrow Transplantation, Karachi, Pakistan; 2 Institute of Experimental Hematology and Transfusion Medicine, Bonn, Germany; 3 University of Sheffield, Sheffield, UK; 4 Children’s Hospital, Lahore, Pakistan; 5 Liaquat university of medical and health sciences, Jamshoro, Pakistan

**Correction to: Thromb J (2017) 15:24**

**https://doi.org/10.1186/s12959-017-0143-3**

Following the publication of this article [1], the authors noted the following typographical errors:

1. Affiliation 3 should read “University of Sheffield, Sheffield, United Kingdom” and Affiliations 6, 7, 8 and 9 were unnecessary duplicates
2. In the abstract the sentence “Ten patients had mutations in FGA followed by three mutations in FGB and three mutations in FGG, respectively” should be “Ten patients had mutations in *FGA* followed by four mutations in *FGB* and two mutations in *FGG*, respectively.”
3. In the Results section the following three sentences:

“In FGA gene, eight mutations were identified as novel and the remaining two were reported mutations. Eight novel mutations include five missense, one nonsense and two frameshift mutations including homozygous and a compound heterozygous frameshift mutation. The two nonsense mutations in FGA are reported in literature. There is one more mutation with reported status in proband (C3). This patient had compound heterozygous mutation with frameshift as novel mutation and nonsense as reported. We identified three mutations in FGB including one novel missense mutation (C9) and two homozygous nonsense mutations reported in siblings. The FGG gene mutations are the rarest of all three fibrinogen genes. We detected three novel mutations including two similar nonsense mutations in siblings and one frameshift mutation in unrelated proband in different exons of FGG gene (Table 1).”

Should be:

“In FGA gene, seven mutations were identified as novel and the remaining three were reported mutations. Seven novel mutations include five missense and two frameshift mutations including homozygous and a compound heterozygous frameshift mutation. The three nonsense mutations in FGA are reported in literature. There is one more mutation with reported status in proband (C3). This patient had compound heterozygous mutation with frameshift as novel mutation and nonsense as reported. We identified four mutations in FGB including one novel missense mutation (C9), two homozygous nonsense mutations reported in siblings and one frameshift mutation(C12). The FGG gene mutations are the rarest of all three fibrinogen genes. We detected two novel similar nonsense mutations in siblings (Table 1).”

4) There are a number of errors in Tables 1 and 2. The corrected versions are provided in this Correction article with the corrections given in bold.
5) Frameshift mutation (p.Gln282Thr fsx83*) and (p. Lys (AAA) 48Arg fs9*) are the novel compound heterozygous mutations which have manifested deletions along with frameshift defects” should in fact read “Frameshift mutations (p.Thr283Arg fs138*) and (p. Lys (AAA) 48Arg fs9*) are the novel compound heterozygous mutations which have manifested deletions along with frameshift defects.

**Table 1:** Genotypic expression of mutations in fibrinogen gene (*FGA, FGB & FGG*)

IP #	Gene	Exon	Mutation	Amino Acid change	Zygosity	Mutation type	Reported/Novel
C1	***FGA***	1	c.24C > A	p.Cys8*	Homozygous	Nonsense	Ref #23 ^€^
C2		2	c.143_144 del AA	p.Lys (AAA)48Arg fs9*	Compound Heterozygous	Frame shift	Novel mutation
C3		5	c.846delG	p.Thr 283Arg fs138*	Compound Heterozygous	Frame shift	Novel mutation
		4	c.385C > T	p.Arg129*	Homozygous	Nonsense	Ref #24 ^€^
C4		4	c.385 C > T	p.Arg129*	Homozygous	Nonsense	Ref #24 ^€^
C5		5	c.598C > T	p.Gln200*	Homozygous	Nonsense	Ref 27*
C6		5	c.904C > G	p.Pro302Ala	Homozygous	Missense	Novel mutation
C7		5	c.913A > G	p.Thr 305 Ala	Homozygous	Missense	Novel mutation
C8		5	c.992A > G	p.Thr331Ala	Homozygous	Missense	Novel mutation
C9^**i**^		5	c.992A > G	p.Thr331Ala	Homozygous	Missense	Novel mutation
C10		5	**c.973A** > G	p.Ser325Gly	Homozygous	Missense	Novel mutation
C11A	***FGB***	2	c.141 > T	p.Arg47*	Homozygous	Nonsense	Ref # 25 ^€^
C11B		2	c.141C > T	p.Arg47*	Homozygous	Nonsense	Ref # 25 ^€^
C9 ^**ii**^		8	c.1294T > A	p.Trp 432Arg	Homozygous	Missense	Novel mutation
**C12**		**2**	**c.118_124dupTTCTTCA**	**TTCTTCA**	**Homozygous**	**Frame shift**	**Novel mutation**
C13A	***FGG***	4	c.361A > T	p.Lys121*	Homozygous	Nonsense	Novel mutation
C13B		4	c.361A > T	Lys121*	Homozygous	Nonsense	Novel mutation

Identified novel and reported mutations in three genes of fibrinogen. The letter A and B with patient code designate the sibling status, **i & ii shows mutation identified in same patient but in different genes**, € (repor`ted mutation) c (complimentary deoxyribonucleic acid), A (adenine), T (thymine), C (cytosine), G (guanine), Lys (lysine), Arg (arginine), Tyr (tyrosine), Pro (proline), Trp (tryptophan), Thr (threonine), Gln (glycine), Cys = cystine, fs = frame shift, * stop codon number, *FGA* (fibrinogen Aα-chain gene), *FGB* (fibrinogen Bβ-chain gene), *FGG* (fibrinogen GƔ-chain gene.

**Table 2:** Assessment of coagulation markers and bleeding scores with consanguinity/ethnicity

IP#	Fibrinogen Level (g/l)	Thrombin Time (Sec)	Prothrombin Time (Sec)	Activated partial thromboplastin Time (aPTT) (Sec)	Bleeding Score	Consanguinity	Interfamilial Relation	Ethnic Origin
*C1	0.01	23	> 120	> 180	20	positive	Unrelated	NA
C2	0.02	24	> 120	> 180	21	positive	Unrelated	Punjabi
C3	0	33	> 120	> 180	22	positive	Unrelated	Punjabi
C4	0.1	24	> 120	> 180	17	positive	Unrelated	Urdu Speaking
C5	0.02	31	> 120	> 180	20	positive	Unrelated	Sindhi
C6	0.01	25	> 120	> 180	20	positive	Unrelated	Urdu speaking
C7	0.02	29	> 120	> 180	22	positive	Unrelated	Sindhi
C8	0.0	30	> 120	> 180	20	positive	Unrelated	Sindhi
C9	0.0	32	> 120	> 180	22	positive	Unrelated	Punjabi
C10	0.01	25	> 120	> 180	16	positive	Unrelated	Punjabi
C11A	0.02	28	> 120	> 180	18	positive	**	Punjabi
C11B	0.01	24	> 120	> 180	16	positive		Punjabi
C12	0.0	30	> 120	> 180	21	positive	Unrelated	Punjabi
C13A	0.02	24	> 120	> 180	20	positive	**	Punjabi
C13B	0.01	25	> 120	> 180	21	positive		Punjabi

Shows the individual test values of PT, aPTT and fibrinogen (Clauss Method), consanguinity and the relationship status. Bleeding score calculated, Tosetto et al [26]. ** Siblings, NA = not available, s (seconds). The fibrinogen levels in all patients were found to be equal to or lower than 0 .1g/l (Normal Range 2-4 g/dl), PT more than 120 s (Normal Range 9–11 s) aPTT more than 180 s (Normal Range 24–27 s) and prolonged thrombin time (normal range 10–13 s). Ethnicity explains the frequency of majorly affected, thickly populated and largest province of Pakistan (Punjab).
